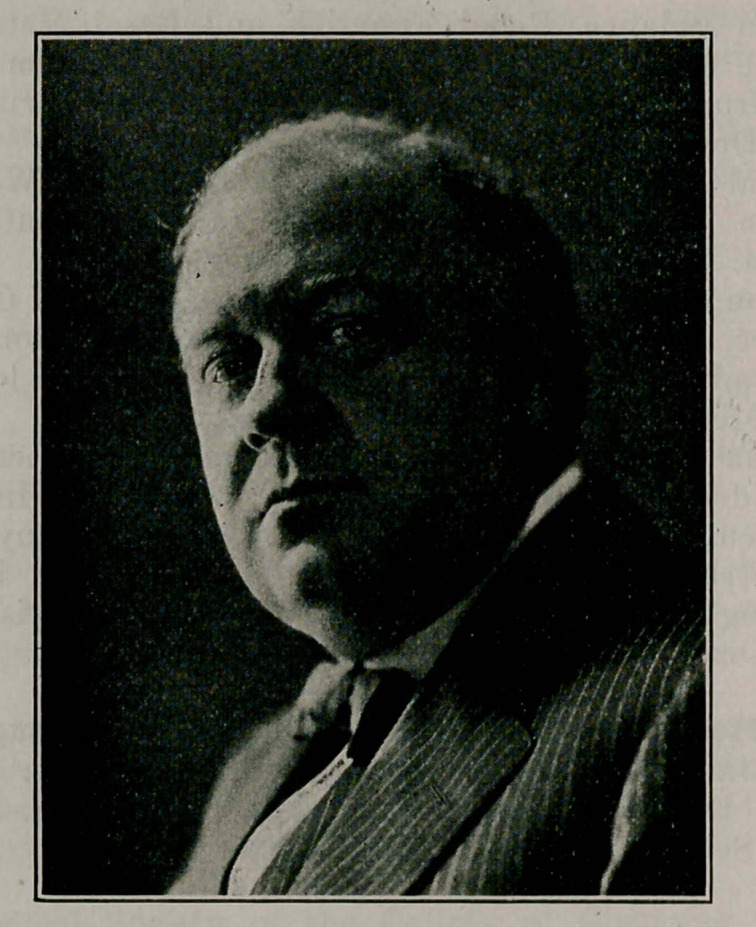# Our Contemporaries

**Published:** 1917-04

**Authors:** 


### OUR CONTEMPORARIES

---

Medicine and Surgery, monthly, $3, makes its debut with the March issue, consisting of 128 pages devoted to surgery. The Editor-in-Chief is Philip Skrainska. There are 15 associates in various cities. The publication office is in the Metropolitan Bdg., St. Louis. The initial editorial on Surgery and Bad Manners is a somewhat unnecessary apology for the military bruskness of the operating room. The contents are of high grade.

---

The Journal of Urology, monthly, $5, begins with the Feb. issue, 138 pages, under the editorship of Hugh Hampton Young, with an executive committee of 3 and associate staff of 18, listed by the universities with which they are connected. It is published from Baltimore.

---

Dr. Smith Ely Jelliffe has been appointed to the staff of the N. Y. Medical Journal, to fill the vacancy caused by the regrettable death of Dr. Claude L. Wheeler. Dr. Jelliffe was editor of the Medical News for four years before its absorption by the N. Y. Medical Journal and, for the past 15 years, has been the publisher of the Journal of Nervous and Mental

Disease. He was also one of the founders of the Psychoanalytic Review. He is well known as an author and editor. His editorial duties will not interrupt his practice.

---

There are over 200 medical journals published in the U. S., the vast majority being monthlies. These may be divided as follows: 1. Organization journals of the A. M. A. and its state branches; 2. Independent, national journals of general scope; 3. Journals representing specialties; 4. Local journals, like this one. Several of the third and a very few of the fourth class are conducted by committees of general or local medical societies, but there is practically no difference on this point as, on the one hand, such journals require a good deal of personal responsibility while, on the other hand, the nominally independent journals must depend on professional cooperation and cannot gain sufficient circulation to be worth while from the financial standpoint.

Viewing the field as critically and as impartially as possible, it may be said that the best journals, i.e., those that give the most for the subscription price include the Journal of the A. M. A., about half of those of the second group, and about half of the third group. The poorest journals, i.e., those that give the least for the subscription price, include many of the state and local journals and a few of the special ones. This appraisal is not made with reference to scientific standards. A journal of the highest scientific value may and, indeed, usually does, serve the interests of a comparatively small number, while a journal that makes no pretense in this direction may be of the greatest value to a large number of practitioners and ultimately to their patients. Generally speaking, any journal excepting in the third group, would really lose its practical value by attempting a standard in excess of the average knowledge and demands of the average practitioner.

---

Endocrinology, the Bulletin of the Association for the Study of the Internal Secretions, makes its first appearance with the issue of Jan. 1917. It is to be a quarterly at the rate of $5, under the able editorship of Dr. Henry R. Harrower, of Glendale, Calif.

---

Southwestern Medicine, begins publication with the Jan. 1917 issue, merging the New Mexico Medical Journal, the Arizona Medical Journal and the Bulletin of the El Paso Co. (Texas) Medical Society. It is published from Las Cruces, N. M., by an editorial board representing the societies mentioned, Dr. R. E. McBride of Las Cruces being editor-in-chief.

## Figures and Tables

**Figure f1:**